# Evaluation of benefits and harms of surgical treatments for post-radical prostatectomy urinary incontinence: a systematic review and meta-analysis protocol

**DOI:** 10.12688/f1000research.19484.1

**Published:** 2019-07-22

**Authors:** Roselyne Choiniere, Patrick O. Richard, Melanie Morin, Le-Mai Tu, Gordon H. Guyatt, Philippe D. Violette

**Affiliations:** 1 Research Center, University of Sherbrooke, Sherbrooke, Quebec, Canada; 2 Division of Urology, Department of Surgery, University of Sherbrooke, Sherbrooke, Quebec, Canada; 3 School of Rehabilitation, University of Sherbrooke, Sherbrooke, Quebec, Canada; 4 Departments of Health Research Methods, Evidence and Impact (HEI) and Surgery, McMaster University, Hamilton, Ontario, Canada

**Keywords:** meta-analysis, systematic review, urinary incontinence, prostatectomy, protocol

## Abstract

**Background**: Post-radical prostatectomy urinary incontinence (PPI) is a frequent and feared complication that can affect approximately 25% of patients. Between 1 and 10% of patients suffering from PPI will require surgery. The effectiveness of the available surgical interventions has only been compared in a few randomized controlled trials and the available reviews have important limitations regarding both benefits and harms that make them insufficient to inform decision-making. The aim of the study is to provide systematic summaries of benefits and harms of contemporary surgical treatment options for PPI through systematic review and meta-analysis using GRADE methodology and reporting in accord with the PRISMA-P statement.

**Methods**: Studies pertaining to bulking agents, male synthetic slings, compressive balloon systems (ProACT) or artificial urinary sphincters (AUS) used for the treatment of patients suffering from PPI will be included. A systematic search will be conducted using the OVID and PubMED platforms in MEDLINE, Embase and Cochrane databases, and reference lists of relevant reviews and guidelines. Trained independent reviewers will conduct study selection and data extraction. Outcomes will include the number of pads used per day, the 24-h pad weight test, the Patient Global Impression of Improvement (PGI-I) and the Incontinence Quality of Life (IQOL) as possible benefits and the reoperations, the Clavien-Dindo complications and the other reported adverse events as the harms. When possible, pooled analyses will be completed. Risk of bias will be assessed using the CLARITY tools and a new tool for the before-and-after studies without a control group. Finally, study heterogeneity will be assessed, publication bias will be evaluated with funnel plots and quality of evidence rated for each outcome.

**Discussion**: Our study will address patient-important outcomes and will be useful in clinical decision-making as well as identifying key elements for future research.

**Study registration**: PROSPERO: CRD42018073923 05/12/2018

## Introduction

Radical prostatectomy is one of the mainstays of treatment for prostate cancer. However, radical prostatectomy is associated with significant morbidity and complications ^1– 3^. A common and feared complication is persistent post-radical prostatectomy urinary incontinence (PPI) ^4^. Following radical prostatectomy, patients suffering from PPI usually report a gradual continence improvement with conservative measures within 12 months postoperatively ^5, 6^. However, reports show that, 1-year after a radical prostatectomy, up to 25% of patients will suffer from some degree of PPI ^7, 8^. This can have significant impact on quality of life and may influence social relationships, emotional health and physical exercise ^9, 10^.

Conservative treatments including lifestyle modifications, bladder training and pelvic floor physiotherapy are recommended as the first-line therapy, but a significant proportion of patients with PPI will seek surgical treatment in the long term due to persistent PPI ^11^. The historic gold standard operation for PPI is the placement of an artificial urinary sphincter (AUS) ^12^. Indeed, a multicenter population-based Canadian study on 25 346 men showed that 2.6%, 3.8% and 4.8% of patients received AUS or a sling at 5, 10 and 15 years following a radical prostatectomy respectively ^13^. Similarly, a nationwide study using the American College of Surgeons National Cancer Database has shown that the incidence of AUS post-radical prostatectomy varied from 1 to 10% (mean 6%) totalling the use of 4 426 AUS for 79 900 Radical prostatectomies (RPs) annually in the United States ^14^.

In contemporary practice, alternative surgical treatments for PPI include four main interventions: bulking agents, male synthetic slings, compressive balloon systems (also known as ProACT ^TM^) and AUS, the current gold standard.

Choosing the adequate device is a challenge for patients and physicians often due to limited understanding of relative efficacy and harms for each intervention. A Cochrane review attempted to summarize the subject in 2014, but was limited due to the paucity of randomized controlled trials (RCTs), in fact, citing one small trial with only 45 patients. Thus, it provided few meaningful conclusions and minimal clinical guidance ^12, 15^.

In 2017, Chen *et al*. published a systematic review and meta-analysis addressing the subject ^16^. By necessity, these authors included data from observational studies in their assessment and did provide an assessment of efficacy for some procedures for PPI. However, this work remains incomplete in several important aspects and has a limited use in clinical decision-making. First, the authors restricted their search to male synthetic slings and AUS. They did not include the bulking agents and they did not report the compressive balloon systems as a separate category. This limits the information on potential treatment choices patients may consider. Second, the authors limited their inclusion criteria to RCTs and prospective observational studies. Unfortunately, the majority of studies published on this topic have a retrospective design. Consequently, the authors were unable to adequately assess harms due to lack of data. Third, although it is believed that certain patient’s characteristics may impact the outcomes of PPI surgeries such as baseline severity of incontinence and history of pelvic radiotherapy, the authors did not ascertain nor discuss the influence of these characteristics on the treatment efficacy ^17, 18^. Fourth, the authors did not specify the time frames used in the analyses of efficacy which lessens the clinical interpretability of their findings given the expected variation of efficacy, harms and quality of life outcomes over time ^19, 20^. Lastly, the majority of data included was nearly a decade old and may not represent contemporary practice.

Overall, this important knowledge gap limits our ability to engage with our patients and make informed decisions about treatment. Many questions remain unanswered regarding the pertinent trade-offs for each device, specifically the potential impact on continence, quality of life and adverse events. We propose to fill this gap by conducting a systematic review and, where possible, a meta-analysis to provide summaries of patient-important outcomes to inform clinical decision-making.

## Methods

### Registration information

This protocol adheres to the recommendations of the Preferred Reporting Items for Systematic Reviews and Meta-analysis Protocols (PRISMA-P) statement, the Grading of Recommendations, Assessment, Development and Evaluation (GRADE) approach and the Cochrane Handbook methodology for Systematic Reviews of Interventions (Version 5.1.0) ^21– 23^. This protocol was registered in PROSPERO CRD42018073923 on December 5 ^th^, 2018.

### Patient and public involvement

This research was done without patient involvement.

## Eligibility criteria

### Study types

RCTs and observational studies that initially enrolled a minimum of 50 patients suffering from PPI per group will be included in the review. Studies without a comparison group will also be included. Primary studies published after January 1 ^st^, 1997 will be included. If more than one study publishes results from the same cohort, the most recent results will be included. There will be no restrictions based on language or country of origin.

### Participant characteristics

Studies that involved adult men suffering from PPI after radical prostatectomy will be included in the review. There will be no restrictions based on radical prostatectomy surgical technique, prostate cancer stages, baseline severity of PPI and history of prior pelvic radiotherapy or previous failed corrective incontinence surgery.

### Intervention types

Any studies reporting on a surgical intervention meant to cure PPI using either an implantation of a device (i.e. male synthetic slings, compressive balloon systems and AUS) or a bulking agent with or without a comparison group will be considered for inclusion. Studies in which the continence surgery was completed simultaneously with another concomitant intervention (i.e. radical prostatectomy, penile prosthesis or any other intervention) will be excluded.

## Outcome measures

Studies reporting at least one of the following outcomes of benefits or harms will be included. Data obtained with more than 20% lost at follow-up will be excluded from the analysis.

**Benefits**

***Primary outcome***

1. Cure, improvement and failure rates defined by the number of pads per day.
  a. Several definitions of treatment success with the number of pads per day are used by authors. We will analyze results according to the following definitions: patients will be considered as 1) cured, if they wear no pad per day to a maximum of a safety pad or 1 pad per day; 2) improved, if they report a reduction of ≥50% of the number of pads per day and/or wear 2 or fewer pads per day; and 3) treatment failures, if they are not cured or improved and/or are wearing 3 or more pads per day.

***Secondary outcomes***

**Efficacy**

2. Cure, improvement and failure rates at the 24-h pad weight test.
  a. The 24-h pad weight test also has different definitions of treatment success. As such, cure will be defined as 24-h pad weight test of 0 g to <10 g per day. Improvement will be characterized as reduction of ≥50% of urine loss per day. Patients that are neither cured nor improved will be considered as treatment failures.

3. The number of pads per day at follow-up.
4. Mean reduction of weight of urine loss at the 24-h pad weight test from baseline.
5. Mean impression of improvement of incontinence will be evaluated by the Patient Global Impression of Improvement (PGI-I) ^24^.

**Quality of life**

6. Improvement of quality of life reported by validated questionnaires such as the Incontinence-Quality of Life (IQOL), which addresses the patient’s feelings about his condition and quantifies bother on daily activities ^25^.

**Harms and adverse events**

***Primary outcome***

7. Reoperation rates.
  a. Reoperations include all direct causes of a secondary surgical procedure such as surgical revisions, explantation and implantation of a subsequent device.

***Secondary outcomes***

8. Short term perioperative complications as reported according to the Clavien-Dindo classification ^26^.
  a. The grades I and II will be considered as minor complications and grades III, IV and V will be considered as major complications.

9. Long-term adverse events as defined as the rates of revision (secondary operation) and explantation (removal) of the surgical device ^27, 28^.
10. Additional reported adverse events will also be documented.

## Information sources

An extensive and systematic electronic search will be performed for the following databases: MEDLINE via PubMED and The Cochrane Library for the relevant publications published from January 1 ^st^, 1997. In addition, reference lists of relevant articles such as review articles and guidelines will be manually screened for other eligible studies.

## Search strategy

This research will be achieved using specific keywords combinations and Medical Subject Headings (MeSH) terms previously defined by our content expert (LMT) and an experienced research librarian for the different platforms. An example of search strategy string is available as *Extended data* ^29^. The identified publications will be managed with the Zotero 5.0 software and duplicates will be removed.

## Study records

### Data management

Throughout the systematic review, data will be managed with the software Zotero 5.0 and Excel sheets.

### Selection process

1. Title and abstract screening: A first screening will be performed by reviewing the title and abstract of each of the identified publication. All reviewers will undergo training before commencing the screening phase. Training will consist of titles and abstracts screening of 15 articles. Trainees will be considered to have successfully completed training if they obtain a concordance of 90% or higher on the standardized test prepared by the first author (RC). Training will be redone if necessary. This first screening will be completed by independent teams of two reviewers. Disagreements between team members will be resolved by discussion among themselves or by a third reviewer (RC), if required. The remaining selected studies will be used for the next step.
2. Full text screening: A second screening will also be performed by independent reviewers to complete study selection. As in the first screening phase, reviewers will be previously trained for the specific criteria of this second step with a screening test of 15 articles. Trainees will be considered to have successfully completed training if they obtain a concordance of 90% or higher. Training will be redone if necessary. Reviewers will perform a full-read selection and record the main reason for excluding each study in line with the PRISMA Collaboration ^21^. Disagreements will be resolved among reviewers’ teams or by a third reviewer (RC), if required. The selection process will be presented in a PRISMA flow diagram.

### Data collection process

Following study selection, data extraction will be performed on the eligible articles in duplicate. A specific data collection form was be created with Excel software and pilot tested (see *Extended data* ^29^). Trained independent reviewers will execute a thorough read of their assigned articles and complete the form. All extracted data will be reviewed and compared between both reviewers of each team. Disagreements of extracted data between reviewers will be resolved among themselves and, if needed, by a third reviewer (PR), a clinician methodologist.

### Data items

The data collection form is available as *Extended data* ^29^ and consists of 4 main sections:

1. General information,
  First author’s last name, year of publication, country of origin of the first author, study design, number of surgeons and centers, enrollment and intervention period, initial number of patients per intervention group, number of patients with PPI, loss of patients at follow-up, length of follow-up, period between radical prostatectomy and continence intervention,
2. Patient characteristics,
  Age, body mass index, baseline severity of urinary incontinence, history of pelvic irradiation,
3. Intervention and comparison,
  Type of device, name of the device, comparative device,
4. Outcome results for efficacy, quality of life and adverse events before and after the intervention.
  Number of pads per day, 24-h pad weight test, the improvement questionnaire, the quality of life questionnaire and adverse events.

### Risk of bias in individual studies

Risk of bias will be assessed for each individual study using instruments appropriate to study design as described below. Pairs of trained independent reviewers will perform risk of bias assessment. Disagreements will be solved among reviewers and, if needed, by a third reviewer (PR or PV). The risk of bias assessment will be represented in coloured graphs.

Studies will be defined as having a lower risk of bias if they have ≤2 "probably high risk of bias" domains and no "definitely high risk of bias" domain. Studies with 3 or more "probably high risk of bias" domains and/or one or more domains associated with a "definitely high risk of bias" will be considered as having a higher risk of bias. In analysis of pooled estimates, studies with a lower risk of bias will be preferred to represent the overall effect if a statistical difference is found between the subgroup pooled estimates.

### Randomized controlled trials

For RCTs, we will use a modified version of one of the Cochrane Risk of Bias instruments, which ranks risk of biases as definitely high to definitely low risk of bias. This tool addresses the biases associated with allocation sequencing, concealment of allocation, blinding, missing data and selective reporting ^30^.

### Observational studies

Risk of bias of cohort studies will be assessed with the CLARITY risk of bias tools. Patient selection, exposure and outcome assessment, as well as, missing data will be evaluated ^30^.

### Before-and-after studies

We anticipate that much of the evidence may come from before-and-after studies without a control group. This design poses specific challenges in assessing risk of bias. We performed a systematic search of the literature in PubMed, Google Scholar, websites of prominent methodological organizations in evidence-based medicine and relevant systematic reviews to identify appropriate risk of bias instruments for before-and-after studies ^31, 32^. We were not able to identify an instrument that we judged fully satisfactory. Many instruments combined quality and reporting issues with assessment of risk of bias and failed to identify issues relevant to this particular review.

Therefore, we developed a before-and-after risk of bias instrument in two stages. Initially, we applied the domains from well-established risk of bias instruments such as those developed by the CLARITY research group, the ROBINS-I, the National Institute of Health before and after tool and an instrument developed by the Johanna Briggs Institute ^30, 33– 35^. Then, we reduced the number of questions to 4 domains that we believe capture the essential risk of biases items for before-and-after studies ^36^. This risk of bias instrument is available as *Extended data* ^29^.

### Data synthesis

Descriptive data will be used to characterize our study population. Characteristics of interest will consist of age, body mass index and timing between radical prostatectomy and incontinence surgery.

Quantitative analyses for benefits and harms outcomes will be performed according to study design. For RCTs and for cohort studies of comparable populations, we will use a random effects model to calculate the pooled estimates of effect size. These pooled estimates will then be represented in forest plots. For before-and-after studies with no control group, each intervention will be analyzed separately to obtain pooled estimates which will be compared to the AUS in a narrative summary. The studies will be weighted using the inverse variance that incorporates the variance and the number of patients. This method will be used to compensate for varying sample size among the studies to allow larger studies to have more weight in the analysis ^37^. Mean differences for continuous outcomes and proportions for dichotomous outcomes with 95% confidence interval (CI) will be pooled. Continuous outcomes will be the change in the 24-hour pad weight test and the improvement and quality of life questionnaires. Dichotomous outcomes for benefits and harms will be the cure, improved and failed rates of treatment, reoperations rates and Clavien-Dindo complications.

Studies will be pooled by types of intervention: bulking agents, male synthetic slings, compressive balloon systems and AUS. We will provide summary data for each intervention type.

Pooled analyses will be performed according to length of follow-up. For all outcomes, with the exception of the Clavien-Dindo complications, a main analysis will be conducted using data acquired from 6 months to less than 36 months, preferring data from the follow-up closest to 12 months. For our primary outcomes of benefits and harms, a secondary analysis will be performed using the longest data available that has been acquired at 36 months of follow-up or more.

Finally, summary statistics will be provided and a narrative report of the findings will be completed. The meta-analyses will be performed in collaboration with an experienced specialized statistician.

### Dealing with missing data

For results reported without a variance measure, we will use the Wan *et al*. method to calculate missing data ^38^. For continuous outcomes, when the standard deviation (SD) of change from before to after the intervention is not reported, we will use an imputation method from the Cochrane Handbook assuming correlations of 0.1, 0.5 and 0.9 to calculate the SD of change from SD at baseline and SD at assessment followed by sensitivity analyses ^23^.

If possible, data reported in graphs will be extracted when not reported otherwise. In addition, when not specified, we will assume the number of participants at assessment by using the initial number of participants.

### Assessment of heterogeneity

Heterogeneity between studies will be measured using visual inspection of the forest plots and χ ^2^ test.

The I ^2^ statistic will be reported to show the percentage of variability that is due to true differences between studies (heterogeneity) rather than a sampling error (chance). In agreement with the Cochrane and GRADE handbooks, 0 to 40% will be interpreted as ‘’might not be important“, 30 to 60% as ‘’may represent moderate heterogeneity”, 50 to 90% as ‘’may represent substantial heterogeneity and 75 to 100% as ‘’considerable heterogeneity” ^23^.

The best available data will be used to provide accurate evidence summary. Within the GRADE approach, outcomes will be reported regardless of I ^2^ results and plausible sources of heterogeneity will be explored with transparency as described below ^39^.

### Subgroup analysis

Heterogeneity may also be explained by some aspects from the studies such as risk of bias, length at follow-up and patients’ preoperative characteristics.

We will perform subgroup analysis by comparing studies rated as having a higher risk of bias to those having lower risk of bias as a means of investigating heterogeneous results.

We believe another explanation for heterogeneity could be the length of follow-up. Our hypothesis is that efficacy and quality of life outcomes will have better results in the shorter term than in the longer term ^19, 20^. We will perform a subgroup analysis for efficacy and quality of life outcomes comparing results obtained at a shorter term (6 to 12 months) and a longer term (more than 12 months).

Several factors need to be considered before choosing a device. Some patient characteristics may influence efficacy outcomes, such as the cure, improvement and failure rates at the number of pads per day. First, we hypothesize that patients who received pelvic radiotherapy will have worse outcomes than non-irradiated patients due to fibrosis and scarring ^40– 42^. Second, we hypothesize that patients suffering from severe PPI will have worse outcomes following continence surgery than patients suffering from mild and moderate PPI ^43, 44^. If there are data available, subgroup analyses will be performed.

### Publication bias

We will detect reporting bias using funnel plots if more than 10 studies are included in any meta-analysis. An asymmetry test will be performed using the Egger method ^45, 46^.

### Confidence in cumulative evidence

Quality of the body of evidence for each outcome will be assessed by using the GRADE approach ^22^. This approach considers the following factors: study design, limitations in study design and implementation, indirectness of evidence, unexplained heterogeneity or inconsistency of results, imprecision of results and publication bias. The latter factor will be assessed by a funnel plot where applicable. Criteria for applicability are described in *Publication bias* section above.

### Dissemination of information

The results of the study will be submitted for publication to a peer-reviewed journal. We also intend to share the results at relevant national and international conferences.

### Study status

This project is ongoing. Study selection is now completed. Data extraction, risk of bias assessment and data analysis have begun, but are not completed.

## Discussion

Our study is intended to be rigorous, systematic and transparent in agreement with the PRISMA-P statement, the GRADE approach and the Cochrane Handbook. Our criteria are precise and explicit and our searches are comprehensive. Our team is composed of content experts, methodologists, a specialized and experienced librarian and a biostatistician specialized in meta-analyses. Moreover, study selection, data extraction and risk of bias assessment will be performed by trained independent teams of reviewers. Data and disagreements will be reviewed by a third reviewer to address discrepancies.

Nevertheless, we anticipate that our study will have some limitations. First, we anticipate the paucity of RCT data. There is a possibility most data will come from observational studies that may have lower reporting standards and affect the quality of the available evidence. Second, many of the observational studies will probably use a before-and-after study design that presents specific biases. To specifically evaluate the quality of these studies, we had to create a new risk of bias assessment instrument, given the lack of an adequate one in the literature. Lastly, our study results will be based on published data and may not reflect unreported or rarely reported harms. Although, publication bias will be assessed and discussed, the possibility of negative studies not being published cannot be excluded.

Nevertheless, in spite of these limitations, our study addresses patient-important outcomes and will be useful in decision-making. Physicians will be able to consult our review and obtain the best current estimates. Our data will be used in guidelines and recommendations to provide guidance to better practices. Also, this meta-analysis will identify the key points for future research on the subject. Finally, this study will also help prepare and guide a future large-scale RCT.

### Protocol registration

This study protocol is registered in PROSPERO CRD42018073923. Available from: http://www.crd.york.ac.uk/PROSPERO/display_record.php?ID=CRD42018073923

## Data availability

### Underlying data

No data is associated with this article.

### Extended data

Open Science Framework: Evaluation of benefits and harms of surgical treatments for post-radical prostatectomy urinary incontinence: a systematic review and meta-analysis protocol. https://doi.org/10.17605/OSF.IO/PVNWX ^29^

This project contains the following extended data:

- Search Strategy String_RChoiniere.docx (A search strategy string used for one of the databases for the systematic review)
- Data Collection Sheet_RChoiniere.xlsx (The data collection sheet used for data extraction in the study)
- Risk of bias Instrument - Before After studies_RChoiniere.docx (The risk of bias instrument created by the Male Incontinence Research Group for before-after studies)

### Reporting guidelines

Open Science Framework: PRISMA-P checklist for "Evaluation of benefits and harms of surgical treatments for post-radical prostatectomy urinary incontinence: a systematic review and meta-analysis protocol". https://doi.org/10.17605/OSF.IO/PVNWX ^29^

Data are available under the terms of the Creative Commons Zero "No rights reserved" data waiver (CC0 1.0 Public domain dedication).
