# Supplementary material for: Evaluation of benefits and harms of surgical treatments for post-radical prostatectomy urinary incontinence: a systematic review and meta-analysis protocol
Source: F1000Res. 2019 Jul 22;8:1155. [Version 1] doi: 10.12688/f1000research.19484.1 (PMC7689604; doi:10.12688/f1000research.19484.1)
Supplement: Peer review history

# Peer review: Reviewer response for version 1

**DOI:** 10.5256/f1000research.21363.r75395

**Published:** 2020-11-25

**Authors:** Bilal Chughtai, Meenakshi Davuluri

**Affiliations:** 1 Weill Medical College of Cornell University, New York, NY, 10065, USA; 2 Weill Cornell Medical College, New York City, NY, USA

The authors of this article have put forth a well thought out and sound methodology for a systematic review and meta analysis of a very important topic in urology. The authors have laid out clear criteria for studies to be included in this and their rationale for including studies beyond randomized control trials. Given the limited data on surgical treatment options for urinary incontinence, we agree with their study design.

 The authors have outlined a comprehensive list of primary and secondary outcomes, which are appropriate for this type of study. While quantifying the degree of incontinence can be challenging, the authors have proposed good objective measures including number of pads used per day and 24 hour pad weight. Additionally, subjective improvement will be measured with the PGI-I and IQOL, with the IQOL measuring the patient’s perspective of bother, a viewpoint which is critical. Additionally, the authors proposed understanding reoperation rates and adverse events, topics of significant interest to both patients and urologists. Our only recommendation to the authors is to include a functional status of patients undergoing the procedures. It is well understood that to utilize an artificial urinary sphincter, the patients need appropriate dexterity. Therefore, it would be helpful to include this (perhaps as an EGOG status) when capturing patient demographics.

 One of the keys to a successful systematic review and meta analysis is the ability to assess for biases. Well known tools will be utilized by the authors including the Cochrane risk of bias tool and the CLARITY tool. However, for before and after studies, the authors have proposed a novel method of assessing bias by adapting other tools into concise questions. While we applaud the innovative efforts of the authors, there is some concern about utilizing a non-validated modality for assessing bias, particularly for before and after study designs. While we understand the authors’ motivation, we suggest considering a way to validate this new tool prior to implementation to enhance the quality of the systematic review and meta analysis.

 Overall, this is a very strong study design and adheres to many of the standardized recommendations of a systematic review and meta-analysis. We applaud the authors for studying a very important and interesting topic within urologic prosthetics. We look forward to seeing the results of the study.

Is the study design appropriate for the research question?

Yes

Is the rationale for, and objectives of, the study clearly described?

Yes

Are sufficient details of the methods provided to allow replication by others?

Yes

Are the datasets clearly presented in a useable and accessible format?

Yes

Reviewer Expertise:

NA

We confirm that we have read this submission and believe that we have an appropriate level of expertise to confirm that it is of an acceptable scientific standard.

# Peer review: Reviewer response for version 1

**DOI:** 10.5256/f1000research.21363.r52771

**Published:** 2019-09-16

**Authors:** Dean S. Elterman, Thomas De Los Reyes

**Affiliations:** 1 Division of Urology, Department of Surgery, University of Toronto, Toronto, ON, Canada

The proposed protocol for a systematic review and meta-analysis on surgical treatments for post-radical prostatectomy incontinence is an important endeavour given the availability of surgical options beyond the artificial urinary sphincter. The authors appreciate the paucity of prospective studies to date, let alone randomized controlled trials on this topic. Thus, retrospective and observational studies are included in this review.

 The authors have a practical approach in defining cure (primary outcome) as “patients wearing no pads per day to a maximum of a safety pad or 1 pad per day". This definition is more clinically relevant and applicable to daily practice. Their proposed secondary outcomes capture the various endpoints typically used in studies related to incontinence, such as 24-hr pad weights and validated questionnaires. Other data items proposed for collection also encompasses important factors of interest such as baseline severity, BMI, age and history of pelvic radiation to name a few.

 With regards to the proposed data analysis component, the authors have anticipated the need for assessing studies with no control groups. They have outlined the risk of bias instrument that they will be using to assess this issue which was modified from validated instruments and reduced to 4 domains of interest (non-validated). However, I wonder if question #3 of the questionnaire “were outcome assessors blinded and independent to the intervention?”  would be able to discern the risk of bias with the study as I anticipate a large proportion of which would the assessors would not be blinded. Choosing aspects of validated methods to ascertain bias does not guarantee accurate assessment of bias. I don't know if taking a portion of a bias tool and applying it invalidates the utility of the tool.

 I look forward to seeing the results of this undertaking given the potential impact it may have on patient care.

Is the study design appropriate for the research question?

Yes

Is the rationale for, and objectives of, the study clearly described?

Yes

Are sufficient details of the methods provided to allow replication by others?

Yes

Are the datasets clearly presented in a useable and accessible format?

Not applicable

Reviewer Expertise:

Suregon-Investigator in Urology with Graduate degree in Clinical epidemiology and health services research.

We confirm that we have read this submission and believe that we have an appropriate level of expertise to confirm that it is of an acceptable scientific standard.
